# Continuous Renal Replacement Therapy (CRRT) in Children and the Specialized CRRT Team: A 14-Year Single-Center Study

**DOI:** 10.3390/jcm9010110

**Published:** 2019-12-31

**Authors:** Keum Hwa Lee, In Suk Sol, Jung Tak Park, Ji Hong Kim, Jae Won Shin, Mi Rireu Park, Jae Hyun Lee, Yoon Hee Kim, Kyung Won Kim, Jae Il Shin

**Affiliations:** 1 Department of Pediatrics, Yonsei University College of Medicine, Yonsei-ro 50, Seodaemun-gu, C.P.O. Box 8044, Seoul 03722, Korea; AZSAGM@yuhs.ac (K.H.L.); KKKJHD@yuhs.ac (J.H.K.); AGUILERA83@naver.com (J.W.S.); QKRALFM27@yuhs.ac (M.R.P.); LJH89515@yuhs.ac (J.H.L.); 2 Division of Pediatric Nephrology, Severance Children’s Hospital, Seoul 03722, Korea; 3 Institute of Kidney Disease Research, Yonsei University College of Medicine, Seoul 03722, Korea; JTPARK@yuhs.ac; 4 Department of Pediatrics, Severance Hospital, Yonsei University College of Medicine, Seoul 03722, Korea; issolkk0312@gmail.com (I.S.S.); YHKIM@yuhs.ac (Y.H.K.); 5 Department of Pediatrics, Hallym University Chuncheon Sacred Heart Hospital, Sakju-ro 77, Gangwon-do, Chuncheon 24253, Korea; 6 Department of Internal Medicine, Yonsei University College of Medicine, Seoul 03722, Korea; 7 Department of Pediatrics, Gangnam Severance Hospital, Yonsei University College of Medicine, Eonjuro 211, Gangnam-gu, Seoul 06273, Korea

**Keywords:** continuous renal replacement therapy (CRRT), specialized CRRT team (SCT), retrospective study

## Abstract

Continuous renal replacement therapy (CRRT) has been used as an important intervention in critically ill children. Our center has the only specialized CRRT team (SCT) for children in Korea, which consists of pediatric intensivists, a pediatric nephrologist and CRRT-specialized-nurses. This study was a retrospective single-center analysis, including all pediatric patients admitted to the intensive care unit (ICU) of Severance hospital in Korea and received CRRT between 2003 and 2016, grouped as before SCT (group A, *n =* 51) and after SCT (group B, *n =* 212). We obtained the data for sex, age, weight, diagnosis, blood flow rate or type of CRRT machine used, administration of inotropic agents or anticoagulants, and ICU duration before CRRT (hours). A total of 263 patients were included. The age was significantly younger (*p* < 0.001) and blood flow rate was lower (*p* = 0.001) in group B than group A. Vasopressors (*p* < 0.001), continuous veno-venous hemodiafiltration (CVVHDF) (*p* < 0.001), nafamostat mesilate (*p* < 0.001), and extracorporeal membrane oxygenation (ECMO)-CRRT (*p* = 0.004) were more frequently used in group B. Based on our 14-year experience, we conclude that SCT operation could have played an important role in increasing the amount of CRRT utilization.

## 1. Introduction

Acute kidney injury (AKI) is defined according to the elevated plasma creatinine level and decreased urine output [1,2,3,4]. It shows diverse clinical manifestations ranging from asymptomatic, through anuria, to multiple organ dysfunctions [5,6]. The prevalence of AKI has been reported to be about 30%–60% for critically ill patients in an intensive care unit (ICU) [7]. In 2017, Kaddourah et al. reported that AKI developed in 26.9%, and severe AKI (renal replacement therapy required) developed in 11.6% of the children in ICU during the first seven days after ICU admission [8]. They also described that AKI itself may crucial to the associated morbidity and mortality and the mortality rate in severe AKI group could be up to 20% [8].

From this point of view, since continuous renal replacement therapy (CRRT) was first introduced by Kramer et al. in 1977 [9], and pediatric CRRT was first used in 1985 [10]; it has been the most important renal replacement modality in critically ill patients. Although both hemodialysis (HD) and peritoneal dialysis (PD) are established interventions for patients who require renal replacement, CRRT is known to be a more efficient therapy for stabilizing circulatory, acid-base, and electrolyte balance when the patient has unstable vital parameters [11]. 

As well as technological advances, other attempts in worldwide have been made to drive the success of the CRRT in the critical patient with AKI. In adult patients, Ronco et al. firstly emphasized the importance of a multidisciplinary approach, including collaboration between various clinical teams [12,13]. After then, several centers opened a specialized CRRT team (SCT) to manage CRRT [14], and two recent observational studies showed that patients had improved CRRT outcomes after the SCT approach [15,16]. 

Despite several studies that have been published demonstrating that CRRT is a very important therapy for critically ill children so far [17,18,19], there have been no reports about the real efficacy of a SCT in the clinic and managing results. Our center is the biggest tertiary care center in Korea with over 14 years of experience in CRRT and has the only specialized CRRT team (SCT) for children in Korea, which consists of pediatric intensivists, a pediatric nephrologist, and CRRT-specialized nurses. Because the SCT was started in August 2008, the objective of this study was to compare and analyze the factors before and after starting SCT management. 

## 2. Methods

### 2.1. Search Strategy

Medical records of 291 patients were collected from January 1, 2003 to April 30, 2016. Of these, 28 underwent more than one CRRT treatment run. Final analysis was done by removing duplicates and leaving only the longest CRRT run. 

During the study period, patients undergoing CRRT were included and grouped as before SCT (group A) and after SCT (group B). Group A included patients treated from March 2003 to July 2008 and group B those from August 2008 to April 2016. Before SCT, pediatric CRRT was run by occasional operators, but after the SCT began, ICU nurses joined and began to work as the member of SCT. Double-lumen catheters ranging between 6.5 and 13.5 F in diameter (Gambro Healthcare, Lakewood, CO, USA) were inserted into the central veins depending on the child’s age and weight. Polyarylethersulfone hollow-fiber hemofilters (PAES; the Prismaflex^®^ HF20, Gambro Lundia AB, Lund, Sweden) and polyacrilonytrile hollow-fiber hemofilters (① AN69^®^ membrane before the year 2010; the Prismaflex^®^ M-10/60/100, Gambro Lundia AB, Lund, Sweden; ② AN69^®^ ST membrane since the year 2010; the Prismaflex^®^ ST-60/100, Gambro Lundia AB, Lund, Sweden) were used in all patients, depending on the patient’s weight. HF-20 or M-10 were used in children weighing less than 10 kg; ST-60 or M-60 were used in patients weighing 10–20 kg, and ST-100 or M-100 were used in children weighing more than 20 kg. Commercially prepared bicarbonate-buffered hemofiltration replacement fluid (Hemosol B0; Gambro Healthcare, Seoul, Korea; potassium free), was used as a dialysate and replacement fluid. Potassium chloride (KCl) was added if the patient has a risk of hypokalemia (20 mEq KCl mix in the 5L Hemozol^®^ when serum potassium level ranged from 3.6 to 4.5 mEg/L and 40 mEq KCl mix in the 5L Hemozol^®^ when serum potassium level lowered than 3.6 mEg/L). The blood flow rate was set as 5 mL/kg/min [18]. The predilution replacement fluid rate or dialysate rate was set at a rate of 2000 mL/1.73 m^2^/hour [18]. The mode of CRRT was selected from one of the following, depending on the patient’s status of solute imbalance: continuous veno-venous hemofiltration (CVVH), continuous veno-venous hemodialysis (CVVHD), and continuous veno-venous hemodiafiltration (CVVHDF). These were determined by the pediatric nephrologist and pediatric intensivist through in-depth discussion. 

The time to initiate CRRT was decided by the pediatric intensivist, depending on each patient’s clinical condition, such as anuria, oliguria (<0.5 mL/kg/hour), or positive fluid balance, regardless of administration of high doses of diuretics (furosemide more than 1 mg/kg/hour). Anticoagulation was not administered during CRRT initiation; however, our protocol establishes that if the filter was blocked within 12 hours of CRRT initiation, anticoagulation agents such as continuous heparin or nafamostat mesilate infusion via the pre-blood pump port were used. The percentage of fluid overload at CRRT initiation (%FO) was calculated using the following formula [20]:

%FO = (Fluid In − Fluid Out)/(ICU admission weight) × 100%(1)

At the initiation of CRRT, the following data were obtained for all patients: sex, age, diagnosis, underlying patient conditions, blood flow rate, use of inotropic agents, anticoagulants, and hours to starting CRRT. 

### 2.2. Definition of SCT

The SCT is a specialized team of physicians and nurses who perform pediatric CRRT. It includes a pediatric nephrologist, pediatric intensivists, nephrologists (internal medicine), and five CRRT-specialized nurses. The pediatric intensivist oversees the critical care and overall decisions on critically ill children–related problems. The pediatric nephrologist determines the distribution of CRRT machines depending upon the daily status of adult and pediatric inpatients in the ICU in consultation with the pediatric intensivist and the nephrologists for adults. CRRT-specialized nurses undergo a CRRT-specialized training course, which includes the basic principles, practical operations, alarm control, and troubleshooting of CRRT; checking hemodynamic stability and CRRT kit status; and management of CRRT catheter-related problems. They work three shifts daily, and their role is separate from that of ICU bedside nurses and chronic HD nurses. They regularly monitor the hemodynamic status and CRRT-related problems of children undergoing CRRT. They are in contact with the machine company and monitor periodic inspections to prevent problems of the CRRT machine itself. Every month, the members of the SCT have a conference, where they share their clinical experiences and provide educational feedback on pediatric CRRT.

### 2.3. Statistical Analysis

Statistical analyses were performed using the SPSS for Windows version 18.0 (IBM Corp., Armonk, NY, USA). An independent *t*-test was used for continuous variables, and they were expressed as means ± standard deviations. Chi-square test and Fisher’s exact test were used to analyze the categorical variables. All differences were considered statistically significant at *p* < 0.05.

## 3. Results

### 3.1. Characteristics of Patients

The demographics and characteristics of the patients treated with CRRT are presented in Table 1. Two hundred and sixty-three patients were included, 212 in group A and 51 in group B. The overall mean age was significantly lower in group B than in group A (*p* < 0.001). There were significantly more patients in group B, especially those aged 1 to 11 months, 3 to 5 years, and 11 to 14 years (*p* = 0.003, *p* = 0.022, *p* = 0.013, respectively). 

The use of inotropic agents at CRRT initiation was also significantly higher in group B than in group A (*p* < 0.001). The differences in the male-to-female ratio (17/34 versus 87/125) and the mean durations in the ICU before CRRT initiation were not significantly different between the two groups (*p* > 0.05). 

The diagnoses of patients who received CRRT were renal disease (e.g., nephrotic syndrome and hemolytic uremic syndrome (HUS)), malignancy and drug intoxication. Cardiac diseases were significantly higher in group B than in group A (*p* = 0.001), and they negatively affect outcome. Malignancy was the most common underlying disease in both groups (70.6% in group A and 36.3% in group B). In contrast to cardiac disease, malignancy was more frequent in group A than in group B, with a statistically significant difference (*p* < 0.001) (Table 2).

The indications for initiating CRRT are listed in Table 3. The most common indications for CRRT in both groups were oliguria refractory to diuretic treatment (54.9% in group A and 49.5% in group B). Fluid overload and sepsis were marked significantly higher in group B than in group A (*p* = 0.041, *p* = 0.027, respectively).

Table 4 demonstrates a comparison of the laboratory variables between the group A and B. There were statistically significant differences between the groups in white blood cell counts (*p* = 0.022) and platelet counts (*p* < 0.001). Other parameters, including blood urea nitrogen (BUN) and creatinine, had no significant difference (*p* > 0.05).

### 3.2. Technical Characteristics of CRRT

The details of the technical characteristics of CRRT are shown in Table 5. In the univariate analyses, more children in group B significantly received a combination of diffusion and convection (CVVHDF) (*p* < 0.001) and fewer received the diffusion-only CRRT modality (CVVHD) (*p* < 0.001). In both groups, only one patient received the convective modality (CVVH).

A total of 148 (56.3%) patients were initiated on CRRT without anticoagulants. More patients in group B were initiated on CRRT with a single infusion of nafamostat mesilate (*p* < 0.001) and a single dose of heparin (*p* < 0.001) once the hemofilter clotted within 12 hours. When anticoagulation started, six (2.8%) patients in group B were switched from heparin to nafamostat mesylate, and five (2.4%) patients were switched from nafamostat mesilate to heparin, but it was not statistically significant (*p* >0.05). The mean blood flow rate was lower in group B than in group A (*p* = 0.001).

**Figure 1:**
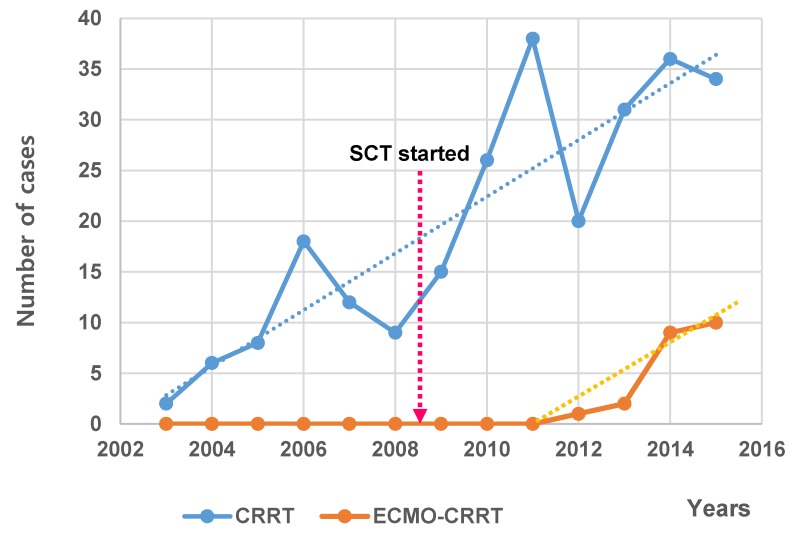
Distribution of CRRT and ECMO-CRRT numbers according to the years. CRRT: continuous renal replacement therapy; ECMO: extracorporeal membrane oxygenation; SCT: specialized CRRT team.

The most common sites of insertion of the initial hemocatheter are the right and left femoral veins in groups A and B, respectively. Since the administration of extracorporeal membrane oxygenation (ECMO)-CRRT to a patient in January 2, 2012, all 27 (12.7%) patients who underwent simultaneous ECMO and CRRT belonged in group B (*p* = 0.004). In most of such cases, the CRRT pump was cannulated into the ECMO circuit. There were only two cases in 2013, including the first case; however, the number of cases had been increasing dramatically over the years following the increase in the rate of CRRT utilization (Figure 1). 

Three different types of CRRT machines were used in pediatric patients: the MultiFiltrate™ CRRT device (FMC), PRISMA^®^ (Gambro Healthcare, Lakewood, CO, USA), and PRISMAFLEX^®^ (Gambro Healthcare). Both PRISMA^®^ and PRISMAFLEX^®^ were more frequently used in group B than in group A, with a statistically significant difference (*p* < 0.001, *p* < 0.001). Only one patient received CRRT with the FMC machine (Table 6). 

Table 7 shows a comparison of the outcomes and parameters between two groups. There were no differences in the duration between CRRT, number of filters used and transfusion during CRRT treatment run, and urine output. Mortality also showed no statistically significance between both groups (*p* > 0.05).

## 4. Discussion

In our study, the increase in the use of CRRT followed the general world trend. In our center, there are about 10 cases of PD, including acute peritoneal dialysis and continuous ambulatory peritoneal dialysis, and about 10 cases of intermittent HD annually. In the same periods in which CRRT data were compared, from March 2003 to July 2008, patients treated with PD and HD numbered 90. On the other hand, from August 2008 to April 2016, patients treated with PD and HD numbered 160. Confirming the general trend, CRRT increased more in group B than group A. The possible reasons are: (1) Before CRRT was developed, only PD could be applied to children, but PD could be dangerous for children in poor condition when inserting PD catheter through the abdomen by major surgery. Compared to PD surgery, hemo-catheter insertion is easier to insert with short duration. (2) We could further consider applying CRRT in ICU because HD has hemodynamic problems in children lighter than 20 kg with unstable vital signs. 

CRRT is an important treatment modality in the field of intensive care. In critically ill children, appropriate treatment has a significant impact on their survival or prognosis. However, research on CRRT has been delayed compared to that on HD or PD worldwide because CRRT must be administered for 24 hours a day, almost all CRRT machines can only be used in the ICU, and a skilled physician is needed to operate these machines. Like elsewhere, in Korea, the numbers of skilled pediatric nephrologists, pediatric intensivists, and well-trained CRRT-specialized nurses are very small, and the number of pediatric patients is much smaller than that of adults. These factors make research on pediatric CRRT difficult. 

According to our results, most patients undergoing CRRT were younger than 10 (73.0%), and there were significantly more infants aged 1 to 11 months and children aged 3 to 5 years after initiating SCT (*p* = 0.003, *p* = 0.022). Furthermore, eight neonates were newly started on CRRT. Young patients develop acute kidney injury (AKI) in the early stage, which might progress rapidly, thus requiring renal replacement. If children develop AKI, they should be carefully monitored in the ICU, and early initiation of CRRT should be considered. The intervention of a SCT exclusively dedicated to pediatric CRRT can avoid the dispersion of knowledge by concentrating the expertise in the hands of few, highly specialized operators, thereby maximally exploiting the information coming from a restricted number of patients. 

The most common positions for catheter insertion were the left and right femoral veins (60.1%). Probably, as the number of younger patients increases, the femoral site is more easily accessible without ultrasonographic guidance. Besides, as the technique improved, entrance into the right and left internal jugular veins, which was previously difficult, became significantly easier after SCT (*p* = 0.036, *p* = 0.029). Along with the worldwide trend, we increased the number of patients treated with CRRT in recent years (see Figure 1). Before SCT, some patients were initiated on CRRT in the emergency room or in the ward rather than in the ICU. The advent of a SCT allowed us a more specific control over a larger number of patients, standardization of procedures, and catheter related problems. Although not measurable, this may have had a positive impact on patient treatment.

For acceptable number of patients, there were more who started CRRT without vasopressors, and less started on vasopressors in group B, indicating, likely, the early use of CRRT (*p* < 0.001). Our study also showed a significant reduction in blood flow rate significantly after the start of the SCT activity (*p* = 0.001). A possible explanation is the improvement of the machine and filter performance, together with the ability of the SCT to cope with the improvement of technology, in order to obtain adequate results with lesser blood flow rates, and consequently, less hemodynamic stress. SCT may allow for improving diagnose the early AKI, giving early application of CRRT, facilitating decision-making and standardized safe extracorporeal therapy. 

Similar to other studies [17,18], the CVVHDF mode was the most common modality used in our study. The initial version of CRRT, which used continuous arteriovenous (AV) filtration modes, is very different from the present one. Because the AV mode is maintained by the patient’s own cardiac output, it showed a high access complication rate; thus, it was not widely applied [21]. Since 2000, after the development of external venovenous circuit pumps, the number of cases using CRRT greatly increased, and it is now a standard therapy for AKI. It effectively resolves fluid retention and improves the electrolyte imbalance simultaneously, especially the CVVHDF mode, which is the most recent modality among the external venovenous modes. In a similar context, the use of PRISMAFLEX^®^, a more specialized CRRT machine for children, also has increased.

There were 27 (10.3%) patients on percutaneous cardiopulmonary support or with ECMO connected to CRRT. In particular, since the first success of ECMO-CRRT in 2012, the rate of ECMO-CRRT utilization has increased rapidly (Figure 1). As mentioned above, this may be due to the fact that the CRRT backup of the SCT has performed well following the development of ECMO technology. The methods of CRRT connection to ECMO, duration of use, and the difference in survival rate were not separately analyzed in this study. Additional studies should be undertaken if plasmapheresis is performed simultaneously with CRRT in patients with liver disease.

The major difference in the use of anticoagulants is that citrate was used in patients with a bleeding tendency in the US study [17], whereas nafamostat mesilate is used in Korea because citrate is not licensed in Korea. In some studies, nafamostat mesilate has been known to have fewer complications, such as hypocalcemia, compared to citrate [22,23]. Because well-trained CRRT-specialized nurses are more efficient in handling nafamostat mesilate, the use of nafamostat mesilate significantly increased after the SCT was established (*p* < 0.001). In Korea, compared with Japan or China, especially in single-group institutions like ours, it is surprising and unusual that nafamostat mesilate has been largely used without notable side effects so far.

Fluid overload or oliguria was an important indication for initiating CRRT in this study. %FO was not significant in the univariate analysis (*p* = 0.348); however, higher %FO indication to CRRT in group B probably reflects an improved sensitization to the %FO importance in outcome determination; i.e., what is expectable in more current times. Although %FO was an important factor to initiate CRRT in the Prospective Pediatric Continuous Renal Replacement Therapy (ppCRRT) Registry study [17], it was not very significant in this study. This could be because the %FO was not accurately measured before and after CRRT. 

The limitations of our study are as follows: Since this was a single-center study, it might have had some bias. Moreover, this was a retrospective study. Over this study period, there were significant changes in the development of machines, technique, and proficiency of the SCT, including the pediatric physicians and CRRT-specialized nurses. In addition, although the number of patients with CRRT increased, it could not be associated with SCT because of other factors, such as increased number of beds in ICU, CRRT machines, and critically ill patients in our center compared to other hospitals. Moreover, univariate analysis of both neonates and 1 to 2-year-old infants showed no statistical significance (*p =* 0.361, *p =* 0.082), but no accurate reason for this could be identified. This could be due to a bias in the process of collecting data and selecting patients.

Nevertheless, Severance Hospital is one of the five major hospitals in Korea, and the number of patients who underwent CRRT in this single center is almost similar to the number of patients in 13 major hospitals in the US. In addition, the study period was relatively longer than that of the ppCRRT (14 versus 5 years), reflecting almost all of the cases of pediatric CRRT in Korea. Moreover, since this was a study of Korean children, it also reflects specific ethnic and national characteristics compared with the US studies. The application of SCT to pediatric patients under the collaboration of pediatric nephrologists, pediatric intensivists, and CRRT-specialized nurses has not been reported worldwide and has never been reported in the US. 

There have been no multicentric CRRT studies in pediatric patients worldwide, apart from the US ppCRRT. Since 2005, when ppCRRT was implemented, there has been no significant research on pediatric CRRT. Based on this study, an effort should be made in Korea to design an index to predict the mortality and increase the survival of patients. 

## 5. Conclusions

The creation of a homogenous group with common tasks, interests, and devotion is essential in managing pediatric CRRT. This is the first study to have identified that the SCT and the organic collaboration of pediatric nephrologists, pediatric intensivists, and CRRT-specialized nurses could have widened the indication of CRRT for critically ill children with AKI and improved management of our patients. 

Although CRRT is used as a first-line treatment for severe AKI in developed countries, it has not yet been introduced in less developed countries. In this global point of view, thorough CRRT training, introduction, and application for physicians are necessary. Further prospective studies will be necessary to evaluate the additional factors for sensitive markers of a SCT efficacy.

## Figures and Tables

**Table 1:** Characteristics of patients receiving continuous renal replacement therapy (CRRT).

Variables	Number of Patients (%)		*p*-Value
	Group A (*n* = 51)	Group B (*n* = 212)	
**Age**	5.0 ± 0.0	3.01 ± 0.21	<0.001
<1 month	0 (0.0%)	8 (3.8%)	0.361
1–11 month	1 (2.0%)	37 (17.5%)	0.003
1–2 year	5 (9.8%)	43 (20.3%)	0.082
3–5 year	15 (29.4%)	33 (15.6%)	0.022
6–10 year	12 (23.5%)	38 (17.9%)	0.360
11–14 year	14 (27.5%)	28 (13.2%)	0.013
15–18 year	4 (7.8%)	25 (11.8%)	0.419
**Sex**			0.312
Male	17 (33.3%)	87 (41.0%)	0.312
**Vasopressors at CRRT initiation**			<0.001
0	14 (27.5%)	154 (72.6%)	<0.001
>1	37 (72.5%)	58 (27.4%)	<0.001
**ICU duration before CRRT (hours)**	6.72 ± 6.39	5.78 ± 6.80	0.476

CRRT: continuous renal replacement therapy; ICU: intensive care unit.

**Table 2:** Underlying diseases of patients receiving CRRT.

Parameter	Number of Patients (%)		*p*-Value
	Group A (*n* = 51)	Group B (*n* = 212)	
**Neurologic disease**	5 (9.8%)	43 (20.3%)	0.112
**Cardiac disease**	2 (3.8%)	31 (14.6%)	0.001
**Renal disease**	3 (5.8%)	16 (7.5%)	1.000
Nephrotic syndrome	1 (1.9%)	2 (0.9%)	1.000
Obstructive uropathy	0 (0.0%)	2 (0.9%)	1.000
HUS	1 (1.9%)	3 (1.4%)	0.580
Rhabdomyolysis	0 (0.0%)	4 (1.9%)	1.000
Denys-Drash syndrome	1 (1.9%)	1 (0.5%)	0.351
AKI on CKD ^†^	0 (0.0%)	4 (1.9%)	1.000
**Liver disease**	1 (2.0%)	20 (9.4%)	0.088
**Malignancy**	36 (70.6%)	77 (36.3%)	<0.001
No tumor lysis syndrome	35 (68.7%)	72 (34.0%)	<0.001
Tumor lysis syndrome	1 (1.9%)	5 (2.3%)	1.000
**Drug intoxication**	0 (0.0%)	1 (0.5%)	1.000
**Pulmonary disease**	1 (2.0%)	11 (5.2%)	0.471
**Metabolic disease**	1 (2.0%)	1 (0.5%)	0.351
**Immune deficiency**	0 (0.0%)	1 (0.5%)	1.000
**Sepsis**	1 (2.0%)	11 (5.2%)	0.471
**Other**	1 (2.0%)	0 (0.0%)	0.194

CRRT: continuous renal replacement therapy; HUS: hemolytic uremic syndrome; AKI: acute kidney injury; CKD: chronic kidney disease. ^†^ Patients who are CKD stage 3–5, not depending upon the cause of CKD. These patients received CRRT due to aggravation of AKI.

**Table 3:** Indications of initiating CRRT.

Indication	Total Number of Patients (%) *		*p*-Value
	Group A (*n* = 51)	Group B (*n* = 212)	
Oliguria	28(54.9%)	105(49.5%)	0.491
Fluid overload	28(54.9%)	83(39.2%)	0.041
Uremia	27(52.9%)	96(45.3%)	0.325
Metabolic acidosis	3(5.9%)	30(14.2%)	0.156
Electrolyte imbalance	8(15.7%)	20(9.4%)	0.208
Sepsis	9(17.6%)	16(7.5%)	0.027
Others ^†^	1(2.0%)	8(3.8%)	1.000

CRRT: continuous renal replacement therapy. * Duplicates are allowed. ^†^ Other indications contain kidney transplantation, applied immediately after continuous ambulatory peritoneal dialysis catheter insertion, rhabdomyolysis, operation, etc.

**Table 4:** Laboratory results of patients receiving CRRT.

Parameter	Group A (*n* = 51)	Group B (*n* = 212)	*p*-Value
**Complete blood count**			
WBC (/mm^3^)	6584.78 ± 8331.47	12822.82 ± 12368.29	0.022
Hemoglobin (g/L)	9.32 ± 2.53	9.63 ± 2.31	0.569
Hematocrit (%)	26.83 ± 7.52	29.33 ± 7.53	0.147
Platelet count (×10^3^/µL)	81.30 ± 61.36	160.84 ± 149.86	<0.001
**Coagulation tests**			
Prothrombin Time (s)	30.53 ± 35.66	28.14 ± 29.32	0.737
aPTT (s)	71.00 ± 50.98	72.87 ± 55.14	0.884
**ABGA**			
pH	7.33 ± 0.17	7.27 ± 0.18	0.147
pCO_2_ (mmHg)	44.21 ± 26.20	41.98 ± 25.57	0.704
pO_2_ (mmHg)	92.20 ± 73.53	99.97 ± 67.20	0.619
Lactate (mg/dL)	4.20 ± 0.00	7.03 ± 5.90	0.634
**Routine chemistry**			
Glucose (mg/dL)	135.32 ± 103.59	144.63 ± 82.83	0.644
Potassium (mg/dL)	4.11 ± 1.18	4.43 ± 1.50	0.342
tCO_2_ (mg/dL)	20.27 ± 8.09	17.29 ± 7.17	0.081
BUN (mg/dL)	32.60 ± 18.43	39.21 ± 38.54	0.433
Creatinine (mg/dL)	1.53 ± 1.30	1.97 ± 2.95	0.497

WBC: white blood cell; aPTT: activated partial thromboplastin time; ABGA: arterial blood gas analysis; pH: acidity; pCO_2_: partial pressure of carbon dioxide; pO_2_: partial pressure of oxygen; tCO_2_: total carbon dioxide; BUN: blood urea nitrogen.

**Table 5:** Technical characteristics of CRRT.

Characteristics	Number of Patients (%)		*p*-Value
	Group A (*n* = 51)	Group B (*n* = 212)	
**Modality**			<0.001
CVVH	0 (0.7%)	1 (0.5%)	1.000
CVVHD	47 (92.2%)	17 (8.0%)	<0.001
CVVHDF	4 (7.8%)	194 (91.5%)	<0.001
**Anticoagulation**			<0.001
No anticoagulation	29 (56.9%)	119 (56.1%)	0.925
Heparin	19 (37.2%)	22 (10.4%)	<0.001
Nafamostat mesilate	2 (3.9%)	60 (28.3%)	<0.001
Nafamostat mesilate → Heparin	0 (0.0%)	6 (2.8%)	0.600
Heparin → Nafamostat mesilate	1 (2.0%)	5 (2.4%)	1.000
**Initial catheter position**			<0.001
Left femoral	13 (25.5%)	70 (33.0%)	0.299
Right femoral	21 (41.2%)	54 (25.5%)	0.026
Right internal jugular	1 (2.0%)	26 (12.3%)	0.036
Left internal jugular	0 (0.0%)	19 (9.0%)	0.029
Subclavian	7 (13.7%)	16 (7.5%)	0.131
ECMO (PCPS)	0 (0.0%)	27 (12.7%)	0.004
No information *	9 (17.6%)	0 (0.0%)	<0.001
**Blood flow rate (mL/min)**	89.86 ± 36.13	70.64 ± 29.59	0.001
Range	30–180	15–120	0.001

CRRT: continuous renal replacement therapy; CVVH: continuous veno-venous hemofiltration. CVVHD: continuous veno-venous hemodialysis; CVVHDF: continuous veno-venous hemodiafiltration ECMO: extracorporeal membrane oxygenation; PCPS: percutaneous cardiopulmonary support. * There was no information about catheter position in the patient chart.

**Table 6:** Distribution of CRRT machines which used to patients.

CRRT Machines	Number of Patients (%)		*p*-Value
	Group A (*n =* 51)	Group B (*n =* 212)	
PRISMA ^®^	51 (100.0%)	166 (78.3%)	<0.001
PRISMAFLEX ^®^	0 (0.0%)	45 (21.2%)	<0.001
FMC ^®^	0 (0.0%)	1 (0.5%)	1.000

CRRT: continuous renal replacement therapy.

**Table 7:** Comparison of outcomes and parameters between the groups in patients receiving CRRT.

Valuables	Group A (*n* = 51)	Group B (*n* = 212)	*p*-Value
Duration of CRRT ± SD (days)	6.72 ± 6.39	5.85 ± 6.83	0.508
Number of filter use during CRRT ± SD (*n*) ^†^	3.67 ± 3.66	4.79 ± 4.39	0.091
Number of TF during CRRT ± SD (*n*) ^†^	0.53 ± 1.50	0.20 ± 0.59	0.133
%FO at CRRT (%)	5.97 ± 7.61	7.35 ± 8.40	0.348
Urine output rate at CRRT (mL/kg/h)	1.24 ± 1.53	1.49 ± 1.80	0.427
CRRT mortality, *n* (%)	34 (66.7%)	150 (70.8%)	0.863

CRRT: continuous renal replacement therapy; TF: transfusion; %FO: percent of fluid overload; SD: standard deviation. ^†^ Number of filters used and TFs during CRRT were counted per patient.
